# Impact of sarcopenia on chemotherapy‐triggered exacerbation of interstitial lung disease in patients with non‐small cell lung cancer

**DOI:** 10.1111/1759-7714.14294

**Published:** 2021-12-28

**Authors:** Ryota Kikuchi, Hiroyuki Takoi, Mayuko Ishiwari, Kazutoshi Toriyama, Yuta Kono, Yuki Togashi, Shinji Abe

**Affiliations:** ^1^ Department of Respiratory Medicine Tokyo Medical University Hospital Tokyo Japan

**Keywords:** exacerbation, interstitial lung disease, non‐small cell lung cancer, prognosis, sarcopenia

## Abstract

**Background:**

While recent evidence has suggested that sarcopenia could predict chemotoxicity, its association with chemotherapy‐triggered interstitial lung disease (ILD) exacerbations has yet to be investigated. Thus, the present study sought to determine whether sarcopenia could predict ILD exacerbations and overall survival (OS) in patients with ILD‐complicated non‐small cell lung cancer (NSCLC).

**Methods:**

From January 2010 to July 2020, 74 patients with ILD‐complicated NSCLC who received chemotherapy were retrospectively investigated. After categorizing patients according to the presence or absence of sarcopenia based on the psoas muscle index, ILD exacerbation rates and OS were evaluated.

**Results:**

Among the patients in the study, 39 were included in the sarcopenia group. Moreover, 17 (22.9%) patients developed ILD exacerbations, with the sarcopenia and nonsarcopenia groups having an exacerbation rate of 33.3% and 11.4%, respectively (*p* = 0.025). Multivariate analysis identified sarcopenia as an independent predictor of ILD exacerbations (*p* = 0.039). Furthermore, patients with sarcopenia demonstrated a significantly shorter median OS compared to those without the same (9.2 vs. 13.3 months; *p* = 0.029).

**Conclusions:**

Sarcopenia predicted chemotherapy‐triggered ILD exacerbation and OS in patients with ILD‐complicated NSCLC, suggesting its utility in determining treatment approaches.

## Graphical abstract

Psoas major cross‐sectional area / height squared less than 6.36 for men and less than 3.92 for females was defined as sarcopenia. Sarcopenia was a predictor of ILD exacerbation by chemotherapy. The prognosis for the sarcopenia group was worse than that for the nonsarcopenia group. Treatment targeting sarcopenia may be a breakthrough in lung cancer with ILD.

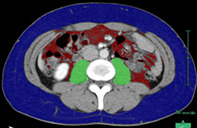

## INTRODUCTION

Follow‐up studies have found that 10%–20% of patients with interstitial lung disease (ILD) had lung cancer.^1^, ^2^ In particular, studies limited to idiopathic pulmonary fibrosis (IPF), which accounts for more than 50% of idiopathic ILD, have shown that 12.7%–31.3% of patients with IPF presented with lung cancer.^1^, ^2^, ^3^, ^4^ Although several recent single‐group trials have investigated patients with lung cancer who have pre‐existing ILD,^5^, ^6^ such patients have largely been excluded from most clinical trials involving chemotherapy development given the risk for treatment associated exacerbation of ILD. Therefore, insufficient evidence is available for the treatment and prognosis of such patients.

Studies have shown that lung cancer with ILD has a poor prognosis because of the risk for chemotherapy‐triggered exacerbation of ILD.^2^, ^7^, ^8^ ILD exacerbation rate at the time of chemotherapy is 27%–34.9%,^9^, ^10^, ^11^ with exacerbation‐related mortality rate ranges from 17.6% to as high as 64.2%.^2^, ^12^, ^13^ Therefore, predicting ILD exacerbation may be beneficial in improving the prognosis of lung cancer with ILD. Although studies have already identified usual interstitial pneumonia patterns in high‐resolution computed tomography (HRCT), low forced vital capacity, Gender‐Age‐Physiology score, and Glasgow prognostic score (GPS) as predictors of exacerbation,^9^, ^10^, ^12^, ^14^, ^15^ clinically effective factors that can reliably predict exacerbations have yet to be elucidated.

Sarcopenia is a syndrome characterized by progressive and systemic loss of skeletal muscle mass and strength.^16^ Incidentally, recent reports have identified low skeletal muscle mass measured via computed tomography (CT) as a poor prognosis factor for lung cancer and ILD.^17^, ^18^, ^19^, ^20^, ^21^, ^22^, ^23^, ^24^, ^25^ Additionally, evidence has shown that sarcopenia is associated with chemotherapeutic dose‐limiting toxicity and severe toxicity events in patients with various cancers.^20^, ^26^, ^27^ Cortellini et al. reported that among patients with lung cancer, those who had sarcopenia exhibited significantly higher hematological toxicity rates compared to those who did not. However, no study has yet investigated the relationship between chemotherapy‐induced lung damage or ILD exacerbation and skeletal muscle mass or sarcopenia.

Although the etiology of sarcopenia remains unclear, chronic inflammation and malnutrition due to regulation of myokine production have been considered causative factors.^16^ Our group have previously reported that GPS, which is calculated using serum C‐reactive protein and albumin, can predict the prognosis and exacerbation of ILD in patients with ILD‐complicated lung cancer,^14^, ^15^ suggesting the potential involvement of inflammation and nutrition in the pathophysiology of this disease. Therefore, the present study sought to determine whether the psoas major muscle area at the third lumbar vertebral level on CT, which has been used as an index that reflects the entire skeletal muscle mass,^28^ could potentially be a biomarker for predicting exacerbation of ILD and overall survival (OS) in patients with ILD‐complicated lung cancer.

## METHODS

### Patients

Consecutive patients with non‐small cell lung cancer (NSCLC) and pre‐existing ILD who received platinum‐based first‐line chemotherapy at Tokyo Medical University Hospital between June 2010 and July 2020 were retrospectively analyzed. This study was approved by the institutional review board of Tokyo Medical University (permission No. TS2020‐0207). This research was conducted in accordance with the 1964 Declaration of Helsinki and amendments. Due to retrospective nature of the study, the need of informed consent was waived by institutional review board of Tokyo Medical University. Our institution's official website had been used as a method to opt out from participation. All patients were cytologically and/or histologically diagnosed with NSCLC. The tumor, node, and metastasis (TNM) stage was evaluated based on the eighth edition of the TNM Classification of Lung Cancer.^29^

ILD was diagnosed when ground‐glass attenuation and reticulation shadow or honeycombing with/without peripheral traction bronchiectasis were observed in both lung fields on chest HRCT. Diagnoses of chemotherapy‐triggered ILD exacerbation were made in accordance with the following criteria detailed in previous studies: (1) deterioration of dyspnea; (2) new bilateral ground‐glass attenuation and/or consolidation superimposed on pre‐existing interstitial shadows; (3) ILD that could not be completely explained by pulmonary infectious disease, heart failure, or fluid overload; and (4) a less than 4‐week interval between the last chemotherapy administration and onset of ILD exacerbation.^9^, ^10^, ^13^, ^14^, ^30^

The following patients were excluded: those (1) whose CT images did not include the spinous process at the third lumbar vertebral level, (2) who had other concomitant active malignancies, infectious diseases, or congested cardiac failure upon initial presentation, (3) who had insufficient clinical data, (4) who had been taking systemic steroids, (5) who had secondary ILD (i.e., interstitial pneumonia associated with connective tissue disease or antineutrophil cytoplasmic antibody‐associated ILD), (6) who had received contraindicated chemotherapeutic drugs in Japan for patients with ILD (i.e., gemcitabine and epidermal growth factor receptor tyrosine kinase inhibitor), and (7) who received thoracic irradiation.

### Data collection

Clinical information and follow‐up data were obtained from patients' medical records. We collected data on patient demographics, age upon first‐line treatment, sex, Eastern Cooperative Oncology Group (ECOG) performance status (PS), TNM stage, histology, body mass index (BMI), laboratory findings, pulmonary function test results, development of exacerbation ILD and mortality data. The follow‐up period lasted until 31 July 2020.

### Image analysis

All patients underwent CT within one month before administration of first‐line chemotherapy. The psoas muscle index (PMI) was measured at the third lumbar vertebral level on CT images using SYNAPSE VINCENT version 5.3.0001 software (Fujifilm Medical Co.) with Hounsfield unit thresholds (−29 to +150) in accordance with previous studies^17^, ^18^, ^19^, ^20^ (Online Resource 1). All CT analyses were independently performed by trained pulmonologists (RK and HT) blinded to the patients' information, after which the obtained data were averaged. The PMI was calculated using the following formula: PMI (cm^2^ /m^2^) = cross‐sectional area of bilateral psoas muscles (cm^2^) / height squared (m^2^). PMI cutoff values for sarcopenia were defined as 6.36 and 3.92 cm^2^/m^2^ for males and females, respectively, based on a previous report defining sarcopenia in Asian adults.^28^

### Statistical analysis

Data were described as numbers (percentages) or median (range). Categorical variables were analyzed using the chi‐square test, whereas continuous variables were compared using the Mann–Whitney's U test. Risk factors for chemotherapy‐triggered exacerbation ILD were identified using univariate and multivariate logistic regression analyses. OS was defined as the time between initial chemotherapy and death or censoring. Patient survival was analyzed using the Kaplan–Meier method, after which differences were compared using the log‐rank test. All statistical analyses were performed using SPSS software version 26.0 (IBM Corp.), with *p* < 0.05 indicating statistical significance.

## RESULTS

### Patient characteristics

Baseline characteristics of the study subjects are provided in Table 1. Among the included patients, 17 (23%) and 57 (77%) were female and male, respectively, with a median age of 71 (range 66–75). Over 90.5% of the patients were diagnosed with an ECOG PS of 0 or 1. Median BMI was 21.6 kg/m^2^ (range 19.0–23.3 kg/m^2^) and 23.8 kg/m^2^ (range 21.5–25.5 kg/m^2^) for female and male patients, respectively. Moreover, 41 patients (55.4%) were histologically diagnosed with adenocarcinoma, whereas 58 patients (78.4%) were diagnosed with stage IV or recurrence. The median serum lactate dehydrogenase and Krebs von den Lungen 6 levels were 221.5 U/l and 681.5 U/ml, respectively. The median predicted forced vital capacity (FVC) and % predicted diffuse capacity of the lung for carbon monoxide were 103.0% and 67.9%, respectively. The median PMI was 4.48 cm^2^/m^2^ (range 3.70–5.43 cm^2^/m^2^) and 6.23 cm^2^/m^2^ (range 5.33–7.14 cm^2^/m^2^) for female and male patients, respectively.

**TABLE 1:** Patient characteristics

	Number (%) or median (range)
Total patients	74 (100%)
Age (years)	71 (66–75)
Sex (%)	
Female	17 (23.0%)
Male	57 (77.0%)
Performance status (%)	
0, 1	67 (90.5%)
2–4	7 (9.5%)
BMI (kg/m^2^)	23.1 (20.9–25.0)
Female	21.6 (19.0–23.3) (*n* = 17)
Male	23.8 (21.5–25.5) (*n* = 57)
Histology (%)	
Adenocarcinoma	41 (55.4%)
Nonadenocarcinoma	33 (44.6%)
Clinical stage (%)	
III	16 (21.6%)
IV or recurrence	58 (78.4%)
LD (U/l)	221.5 (198–269.2)
KL‐6 (U/ml)	681.5 (472.5–1029.5)
% predicted FVC (%)	103.0 (84.8–118.0)
% predicted DLCO (%)	67.9 (53.3–88.2) (*n* = 28)
PMI (cm^2^/m^2^)	5.91 (5.02–6.65)
Female	4.48 (3.70–5.43) (*n* = 17)
Male	6.23 (5.33–7.14) (*n* = 57)

Abbreviations: BMI, body mass index; DLCO, diffuse capacity of the lung for carbon monoxide; FVC, forced vital capacity;^.^KL‐6, Krebs von den Lungen 6;^.^LD, lactate dehydrogenase;^.^PMI, psoas muscle index.

### Correlation between sarcopenia and clinicopathological parameters

Based on the PMI cutoffs for Asian adults, 39 (52.7%) patients were diagnosed with sarcopenia. Table 2 summarizes the baseline clinicopathological characteristics of the patients according to sarcopenia status. Male patients were more likely to have sarcopenia compared to female patients. No significant differences were noted between both groups except for sex (*p* = 0.028), BMI of males (*p* = 0.044), and PMI (*p* < 0.001 and *p* < 0.001 in females and males, respectively).

**TABLE 2:** Clinical characteristics stratified according to sarcopenia status

	Number (%) or median (range)		
	Nonsarcopenia (*n* = 35)	Sarcopenia (*n* = 39)	*p*‐value
Age (years)	69 (64–74)	72 (69–76)	0.054
Sex (%)			
Female	12 (34.3%)	5 (12.8%)	0.028
Male	23 (65.7%)	34 (87.2%)	
Performance status (%)			
0, 1	31 (88.6%)	36 (92.3%)	0.583
2–4	4 (11.4%)	3 (7.7%)	
BMI (kg/m^2^)	23.5 (20.2–25.7)	23.1 (21.0–24.2)	0.258
Female	20.9 (19.1–23.6) (n = 12)	21.6 (17.7–23.3) (*n* = 5)	0.799
Male	24.4 (22.9–26.5) (n = 23)	23.1 (21.0–24.7) (*n* = 34)	0.044
Histology (%)			
Adenocarcinoma	23 (65.7%)	18 (46.2%)	0.091
Nonadenocarcinoma	12 (34.3%)	21 (53.8%)	
Clinical stage (%)			
III	7 (20.0%)	9 (23.1%)	0.748
IV or recurrence	28 (80.0%)	30 (76.9%)	
LD (U/l)	223 (202–267)	220 (197–270)	0.641
KL‐6 (U/ml)	628 (460–1549)	684 (476–963)	0.603
% predicted FVC (%)	101.4 (86.5–114.8)	103.0 (82.8–118.6)	0.984
% predicted DLCO (%)	63.7 (42.3–84.9) (*n* = 12)	70.4 (62.6–96.7) (*n* = 16)	0.260
PMI (cm^2^/m^2^)	6.71 (5.59–7.38)	5.35 (4.62–6.08)	<0.001
Female	4.96 (4.24–5.70) (*n* = 12)	3.44 (2.18–3.70) (n = 5)	<0.001
Male	7.29 (6.71–7.60) (*n* = 23)	5.41 (5.09–6.11) (n = 34)	<0.001

Abbreviations: BMI, body mass index; DLCO, diffuse capacity of the lung for carbon monoxide; FVC, forced vital capacity; KL‐6, Krebs von den Lungen 6; LD, lactate dehydrogenase; PMI, psoas muscle index.

### Exacerbation of chemotherapy‐triggered ILD

Among the 74 patients, 17 (22.9%) were diagnosed with chemotherapy‐triggered exacerbation ILD. Thereafter, risk factors for ILD exacerbation were evaluated (Table 3). Univariate logistic regression analysis showed that the incidence ILD exacerbation was significantly associated with % predicted FVC (odds ratio [OR], 0.96; 95% confidence interval [CI}, 0.94–0.99; *p* = 0.035) and sarcopenia (OR, 3.87; 95% CI: 1.12–13.33; *p* = 0.032). Multivariate analysis identified age (OR, 0.89; 95% CI: 0.80–0.98, *p* = 0.026), % predicted FVC (OR, 0.96; 95% CI: 0.93–0.99; *p* = 0.026), and sarcopenia (OR, 6.39; 95% CI: 1.09–37.26; *p* = 0.039) as significant independent predictive factors for ILD exacerbation after adjusting for age, sex, BMI, % predicted FVC, and sarcopenia.

**TABLE 3:** Factors associated with chemotherapy‐triggered exacerbation of ILD following univariate and multivariate analyses

	Univariate analysis			Multivariate analysis		
Factors	OR	95% CI	*p*‐value	OR	95% CI	*p‐*value
Age, per year increment	0.93	0.86–1.01	0.105	0.89	0.80–0.98	0.026
Sex (Female vs. Male)	0.64	0.18–2.17	0.474	0.71	0.11–4.31	0.712
Performance status (0, 1 vs. 2–4)	1.38	0.24–7.88	0.712			
BMI, per kg/m^2^ increment	0.92	0.77–1.11	0.417	0.98	0.79–1.22	0.921
Histology (adenocarcinoma vs. nonadenocarcinoma)	0.60	0.19–1.86	0.382			
Clinical stage (III vs. IV or recurrence)	1.37	0.34–5.54	0.651			
LD, per U/l increment	1.00	0.99–1.01	0.072			
KL‐6, per U/ml increment	1.00	1.00–1.00	0.353			
% predicted FVC, per % increment	0.96	0.94–0.99	0.035	0.96	0.93–0.99	0.026
% predicted DLCO, per % increment (*n* = 28)	0.73	0.95–1.03	0.993			
Sarcopenia (No vs. Yes)	3.87	1.12–13.33	0.032	6.39	1.09–37.26	0.039

Abbreviations: BMI, body mass index; CI, confidence interval; DLCO, diffuse capacity of the lung for carbon monoxide; FVC, forced vital capacity; KL‐6, Krebs von den Lungen 6; LD, lactate dehydrogenase; ILD, interstitial lung disease; OR, odds ratio.

### Prognosis

**FIGURE 1:**
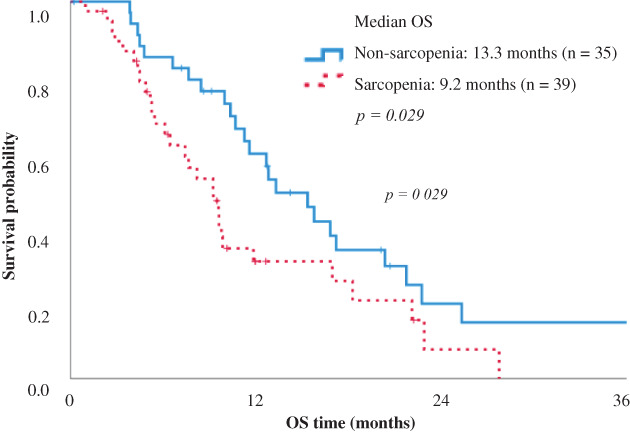
Kaplan–Meier curves showing the overall survival of patients who did (*n* = 39) and did not have sarcopenia (*n* = 35)

Kaplan–Meier analysis was conducted to examine the impact of sarcopenia on survival. Figure 1 shows that those who had sarcopenia demonstrated a significantly worse OS compared to those who did not (9.2 vs. 13.3 months; *p* = 0.029). The chemotherapy regimens are detailed in Online Resource 2. The combination of carboplatin and paclitaxel with/without bevacizumab was the most frequently used first‐line chemotherapy. After comparing chemotherapy regimens between patients who did and did not have sarcopenia, no significant differences were found (Online Resource 3).

## DISCUSSION

To the best of our knowledge, this is the first study to evaluate and explore the impact of sarcopenia in patients with NSCLC and pre‐existing ILD by quantitatively measuring PMI. The present study showed a correlation between sarcopenia and the incidence of ILD exacerbation and prognosis, with sarcopenia having been identified as an independent predictor of chemotherapy‐triggered exacerbation of ILD after adjusting for established prognostic factors of NSCLC and ILD. Furthermore, our findings showed that patients with sarcopenia exhibited a significantly lower median OS compared to those without it. The aforementioned results suggest the importance of monitoring PMI in clinical settings. Furthermore, the management of sarcopenia may prevent exacerbations of ILD and improve prognosis, highlighting a novel therapeutic target among such patients.

Although several studies have investigated the relationship between ILD prognosis and skeletal muscle mass,^21^, ^22^, ^23^, ^24^ only one has thus far examined the relationship between ILD exacerbations and skeletal muscle mass. Awano et al. reported that the cross‐sectional area of the erector spinae muscles measured at the 12th thoracic vertebral body level predicted the prognosis of patients with IPF but did not contribute to IPF exacerbation,^23^ which diffed from the results presented herein. This could have been attributed to our use of the psoas major muscle instead of the erector spinae muscle. Although all previous studies on ILD have used the erector spinae muscles, lung cancer studies often use the psoas major muscle at the third lumbar spine level^17^, ^18^, ^19^, ^20^, ^31^ given that the muscle group found at the cranial level 5 cm from the fourth–fifth lumbar vertebra has been considered the most accurate estimate of whole‐body muscle mass.^32^ The PMI used in the present study, which can be easily calculated from the psoas major muscle cross‐sectional area and height, has been suggested to have a strong correlation with the skeletal muscle index.^28^ Moreover, criteria for low skeletal muscle mass among Asians (6.36 and 3.92 cm^2^ / m^2^ for men and women, respectively) have been widely used in lung cancer research as indicators of sarcopenia.^17^, ^18^, ^31^ The present study, which has been the first to utilize the PMI for ILD, found that sarcopenia can predict chemotherapy‐triggered ILD exacerbation. As such, we believe that sarcopenia evaluation based on the psoas major muscle may become the standard in the future studies investigating ILD.

ILD exacerbations observed herein had been limited to antineoplastic causes, which could also be another point of difference from a previous report.^23^ Although various chemotherapy regimens had been included, patients with ILD who received the contraindicated chemotherapy in Japan were excluded, which would expectedly decrease the potential impact of chemotherapy on the incidence of ILD exacerbation. Furthermore, after grouping the patients according to the presence or absence of sarcopenia, no significant difference in chemotherapy regimens had been noted, suggesting that sarcopenia can predict ILD exacerbations regardless of the type of anticancer drugs administered.

Studies have shown that patients with lung cancer and ILD exhibited poorer prognosis than uncomplicated cases owing to the exacerbation of ILD.^2^, ^7^, ^8^ In this study, sarcopenia predicted not only the onset of exacerbations but also the prognosis. Therefore, the poor prognosis in the sarcopenia group can be attributed to ILD exacerbation. By contrast, reports have also shown no significant association between ILD exacerbation and prognosis in patients with ILD‐complicated lung cancer^10^, ^33^ Patients with lung cancer and ILD have been reported to have a lower disease control rate than uncomplicated cases,^34^ with the poor prognosis perhaps being due to anticancer drug resistance. At present, it is unclear whether suppressing exacerbation caused by chemotherapy will improve the prognosis of lung cancer with ILD, suggesting the need for investigating additional cases.

The reason for the involvement of sarcopenia in the ILD exacerbations and prognosis of lung cancer with ILD remains unclear. However, such involvement may be attributed to myokine, a bioactive substance secreted by skeletal muscle. Peroxisome proliferator‐activated receptor γ coactivator 1α (PGC‐1α), a transcriptional coactivator required for the maintenance and generation of mitochondria, has been identified as an important factor in the relationship between exercise and inflammation.^35^ Patients with sarcopenia have been found to have reduced PGC‐1α in their skeletal muscle but enhanced interleukin*‐*6 and tumor necrosis factor‐α expression, causing inflammation and muscle proteolysis^36^, ^37^ Previous studies have suggested that inflammation and undernutrition may contribute to the prognosis and exacerbation of ILD.^14^, ^38^, ^39^, ^40^, ^41^ Based the aforementioned findings, anti‐inflammatory treatment through myokine supplementation and maintenance of muscle mass through nutrition therapy and rehabilitation may be novel approaches for suppressing ILD exacerbation, as well as improving the prognosis for lung cancer with ILD.

At present, no treatment has been established for ILD exacerbation, which has shown high mortality rates.^2^, ^7^, ^8^ Therefore, patients with lung cancer and ILD often do not receive chemotherapy.^42^, ^43^ On the other hand, given evidence suggesting that chemotherapy may prolong survival, uniformly excluding these populations from chemotherapy may not be appropriate.^5^, ^6^ The present study showed that patients without sarcopenia were less prone to ILD exacerbation and could hence receive aggressive chemotherapy, which may contribute to a better prognosis. On the other hand, patients with sarcopenia may have a worse prognosis due to ILD exacerbation and hence require careful chemotherapy. Thus, the presence or absence of sarcopenia may be useful in determining treatment approaches for patients with lung cancer and ILD. Considering that these patients undergo whole‐body CT for the purpose of staging evaluation, sarcopenia evaluation based on the psoas major muscle can be performed quite easily. Therefore, routine evaluation of sarcopenia upon introducing a chemotherapeutic drug may be better for patients with lung cancer and ILD.

In this study, ILD exacerbation occurred in six patients on first‐line chemotherapy and 11 patients on second to fifth line chemotherapy. Moreover, docetaxel was the most common anticancer drug that caused the exacerbation (Online Resource 4). Studies have shown that patients with lung cancer after chemotherapy have decreased skeletal muscle mass compared with that before chemotherapy.^44^ Therefore, increasing exacerbation at the late phase may have been caused by the large number of patients with sarcopenia. By contrast, given that docetaxel is often used after second‐line chemotherapy, it is possible that the exacerbation period was in the late phase depending on the use of anticancer drugs.

The present study has several limitations worth noting. First, this was a single‐center retrospective study with a relatively small sample size, which may have introduced bias. However, lung cancer with ILD is a rare disease with a limited number of patients^3^, ^4^, ^9^, ^10^, ^12^, ^13^, ^14^, ^42^, ^45^ The number of patients included herein does not considerably differ from those included in other studies. Moreover, given the absence of large‐scale phase III trials in patients with ILD‐complicated lung cancer to date, evidence has largely been obtained from retrospective studies. Therefore, despite these limitations, we believe that the results presented herein are of considerable significance. Further studies are required to confirm our observations in other external validation cohorts. Second, diseases (e.g., diabetes, osteoporosis), drugs (e.g., statins, sulfonylureas, glinides, and antifibrotic drugs), and lifestyles (e.g., exercise and diet) that may affect skeletal muscle mass could not be completely assessed. On the other hand, the effects of sarcopenia due to complications can be considered minimal given that patients who had received steroids, had malignant tumors other than lung cancer, and had secondary ILD were excluded. Third, although the total psoas area was used, it might have been better to measure whole‐body skeletal muscle mass using bioelectrical impedance or dual‐energy X‐ray absorption. However, these devices expose the patients to radiation and are considerably costly. Therefore, CT can be considered appropriate from the viewpoint of convenience.

The present study showed that sarcopenia can predict ILD exacerbations and OS in patients with ILD‐complicated NSCLC who received chemotherapy. In the future, targeting sarcopenia can be expected to become a novel approach for suppressing ILD exacerbation and improving patient prognosis.

## CONFLICT OF INTEREST

The authors declare no competing interests.

## Supporting information


**Online Resource 1** Bilateral psoas major muscle (green area) at the third lumber vertebral level

**Online Resource 2** Frequency of chemotherapy regimens during the clinical course

**Online Resource 3** Frequency of first‐line chemotherapy regimens stratified according to sarcopenia status

**Online Resource 4** Number of chemotherapy‐triggered exacerbation of ILD in each chemotherapy regimen
