# Supplementary figures and images for: Impact of sarcopenia on chemotherapy‐triggered exacerbation of interstitial lung disease in patients with non‐small cell lung cancer
Source: Thorac Cancer. 2021 Dec 28;13(4):549–56. doi: 10.1111/1759-7714.14294 (PMC8841712; doi:10.1111/1759-7714.14294)

**Online Resource 1.** Bilateral psoas major muscle (green area) at the third lumber vertebral level


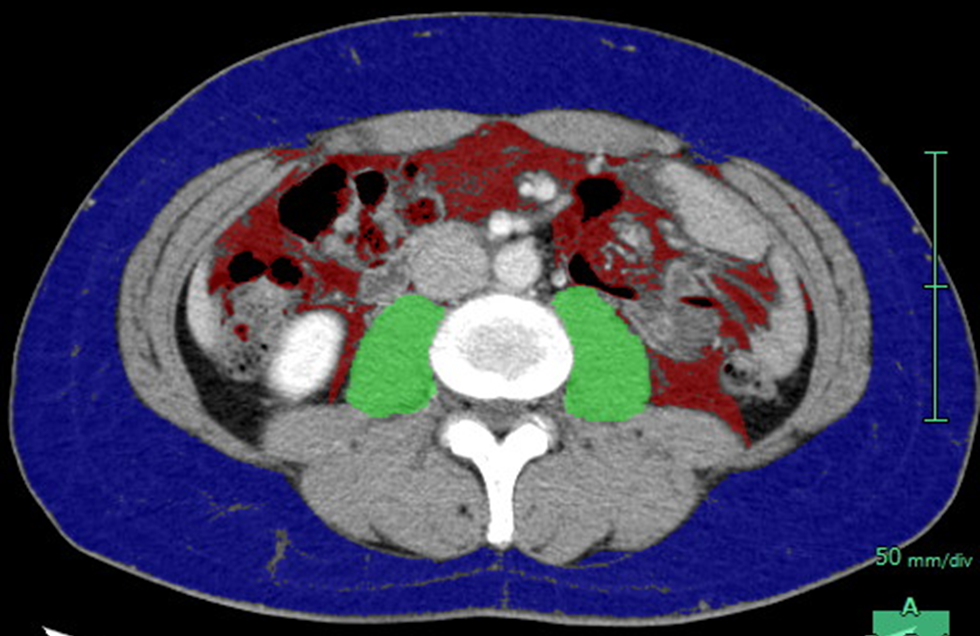

Supplement: Supplementary file 1 — Online Resource 1 Bilateral psoas major muscle (green area) at the third lumber vertebral level [file TCA-13-549-s002.docx]
