# Supplementary material for: Impact of sarcopenia on chemotherapy‐triggered exacerbation of interstitial lung disease in patients with non‐small cell lung cancer
Source: Thorac Cancer. 2021 Dec 28;13(4):549–56. doi: 10.1111/1759-7714.14294 (PMC8841712; doi:10.1111/1759-7714.14294)
Supplement: Supplementary file 2 — Online Resource 2 Frequency of chemotherapy regimens during the clinical course [file TCA-13-549-s001.docx]

**Online Resource 2**. Frequency of chemotherapy regimens during the clinical course

| **First line**  **(n = 74)** | **N** | **Second line**  **(n =46)** | **N** | **Third line**  **(n = 16)** | **N** | **Fourth line**  **(n = 6)** | **N** | **Fifth line**  **(n = 3)** | **N** |
| --- | --- | --- | --- | --- | --- | --- | --- | --- | --- |
| CBDCA + PTX with/without BEV | 39 | DOC | 21 | DOC  +RAM | 3 | Nivo-  lumab | 2 | S1 | 1 |
| CDDP/CBDCA + PEM with/without BEV | 18 | PEM | 12 | DOC | 3 | VNR | 1 | Nivo-  Lumab | 1 |
| CBDCA  + Nab-PTX | 10 | S1 | 5 | Nivo-  lumab | 3 | Nab-  PTX | 1 | DOC | 1 |
| CBDCA + S1 | 4 | Pembro-  lizumab | 3 | PEM | 2 | DOC | 1 |  |  |
| CDDP/CBDCA + VNR | 2 | CBDCA  +ETP | 2 | S1 | 2 | S1 | 1 |  |  |
| CDDP + ETP | 1 | Nivo-  lumab | 1 | Nab-  PTX | 1 |  |  |  |  |
|  |  | VNR | 1 | VNR | 1 |  |  |  |  |
|  |  | CBDCA  +PTX | 1 | Atezo-  lizumab | 1 |  |  |  |  |

N, number; CBDCA, carboplatin; PTX, paclitaxel; BEV, bevacizumab; CDDP, cisplatin; PEM, pemetrexed; Nab-PTX, nanoparticle albumin-bound paclitaxel; VNR, vinorelbine;

ETP, etoposide; DOC, docetaxel; RAM, ramucirumab
