# Supplementary material for: Impact of sarcopenia on chemotherapy‐triggered exacerbation of interstitial lung disease in patients with non‐small cell lung cancer
Source: Thorac Cancer. 2021 Dec 28;13(4):549–56. doi: 10.1111/1759-7714.14294 (PMC8841712; doi:10.1111/1759-7714.14294)
Supplement: Supplementary file 3 — Online Resource 3 Frequency of first‐line chemotherapy regimens stratified according to sarcopenia status [file TCA-13-549-s004.docx]

**Online Resource 3.** Frequency of first-line chemotherapy regimens stratified according to sarcopenia status

|  | Number (%) | | *p*-value |
| --- | --- | --- | --- |
|  | No sarcopenia | Sarcopenia |  |
| **Regimens** | 35 | 39 |  |
| CBDCA + PTX  with/without BEV | 18 (51.4%) | 21 (53.8%) | 0.835 |
| CDDP/CBDCA + PEM  with/without BEV | 9 (25.7%) | 9 (23.0%) | 0.792 |
| CBDCA + nab-PTX | 5 (14.7%) | 5 (12.8%) | 0.854 |
| Others | 3 (8.5%) | 4 (10.2%) | 0.805 |

CBDCA, carboplatin; PTX, paclitaxel; BEV, bevacizumab; CDDP, cisplatin; PEM, pemetrexed; Nab-PTX, nanoparticle albumin-bound paclitaxel. Others: CBDCA + S1 (n = 4), CBDCA + vinorelbine (n = 2), CBDCA + etoposide (n = 1).
