# Supplementary material for: Impact of sarcopenia on chemotherapy‐triggered exacerbation of interstitial lung disease in patients with non‐small cell lung cancer
Source: Thorac Cancer. 2021 Dec 28;13(4):549–56. doi: 10.1111/1759-7714.14294 (PMC8841712; doi:10.1111/1759-7714.14294)
Supplement: Supplementary file 4 — Online Resource 4 Number of chemotherapy‐triggered exacerbation of ILD in each chemotherapy regimen [file TCA-13-549-s003.docx]

**Online Resource 4**. Number of chemotherapy-triggered exacerbation of ILD in each chemotherapy regimen

| **Chemotherapy** | **Number** |
| --- | --- |
| DOC | 9 |
| CBDCA + Nab-PTX | 2 |
| CBDCA + PTX + BEV | 1 |
| CDDP + PEM | 1 |
| CBDCA + PEM | 1 |
| PEM | 1 |
| CBDCA + PEM + BEV | 1 |
| Nivolumab | 1 |

ILD, interstitial lung disease; DOC, docetaxel; CBDCA, carboplatin;

Nab-PTX, nanoparticle albumin-bound paclitaxel; PTX, paclitaxel; BEV, bevacizumab; CDDP, cisplatin; PEM, pemetrexed.
